# Medicaid Expansion Under the Affordable Care Act and Early Mortality Following Lung Cancer Surgery

**DOI:** 10.1001/jamanetworkopen.2023.51529

**Published:** 2024-01-12

**Authors:** Leticia M. Nogueira, Daniel J. Boffa, Ahmedin Jemal, Xuesong Han, K. Robin Yabroff

**Affiliations:** 1 Surveillance and Health Equity Science, American Cancer Society, Atlanta, Georgia; 2 Division of Thoracic Surgery, Yale School of Medicine, New Haven, Connecticut

## Abstract

**Importance:**

Medicaid expansion under the Patient Protection and Affordable Care Act is associated with gains in health insurance coverage, earlier stage diagnosis, and improved survival among patients with cancer.

**Objective:**

To examine the association of Medicaid expansion with changes in early mortality among adults undergoing surgical resection of non–small cell lung cancer (NSCLC), a setting in which access to care is a major determinant of survival.

**Design, Setting, and Participants:**

This cohort study used the National Cancer Database to identify 14 984 adults 45 to 64 years of age who underwent surgical resection of NSCLC between 2008 and 2019. Analysis was conducted between March 28, 2021, and September 1, 2023.

**Exposure:**

State of residence Medicaid expansion status.

**Main Outcomes and Measures:**

Descriptive statistics were used to compare study population characteristics by Medicaid expansion status of patients’ state of residence. Difference-in-differences analyses were used to evaluate the association between Medicaid expansion and postoperative mortality before implementation of the ACA (2008-2013) vs after (2014-2019).

**Results:**

Among 14 984 adults included, the mean (SD) age was 56.3 (5.1) years, 54.6% were women, and 62.1% lived in Medicaid expansion states. Both 30-day (from 0.97% to 0.26%) and 90-day (from 2.63% to 1.32%) postoperative mortality decreased from before the ACA to after among patients residing in Medicaid expansion states (both *P* < .001) but not in nonexpansion states (30-day mortality before the ACA, 0.75% vs after the ACA, 0.68%; *P* = .74; and 90-day mortality before the ACA, 2.43% vs after the ACA, 2.20%; *P* = .57), leading to a difference-in-differences of −0.64 percentage points (95% CI, −1.19 to −0.08; *P* = .03) for 30-day mortality and −1.08 percentage points (95% CI, −2.08 to −0.08; *P* = .03) for 90-day mortality. The difference-in-differences for in-hospital mortality was not significant (*P* = .34) between expansion states (1.41% before the ACA to 0.77% after the ACA; 0.63 percentage point decrease; *P* = .004) and nonexpansion states (1.49% before the ACA to 1.20% after the ACA; 0.30 percentage point decrease; *P* = .29).

**Conclusions and Relevance:**

In this cohort study of patients with NSCLC, Medicaid expansion was associated with declines in 30- and 90-day postoperative mortality following hospital discharge. These findings suggest that Medicaid expansion may be an effective strategy for improving access to care and cancer outcomes in this population.

## Key Points

**Question:**

Is Medicaid expansion under the Patient Protection and Affordable Care Act associated with improvement in postoperative survival among patients with non–small cell lung cancer (NSCLC)?

**Findings:**

In this cohort study assessing 14 984 adults 45 to 64 years of age with NSCLC identified in the National Cancer Database, Medicaid expansion was associated with a significant reduction in 30- and 90-day mortality after discharge from the hospital following surgical resection of stage I to III NSCLC.

**Meaning:**

These results suggest that during a period when access to care is crucial (ie, recovery from major surgery), Medicaid expansion may be associated with improved survival.

## Teaser

This nationwide cohort study assess whether Medicaid expansion under the Patient Protection and Affordable Care Act is associated with changes in early mortality among adults after surgical resection of non–small cell lung cancer.

## Introduction

Medicaid expansion under the Patient Protection and Affordable Care Act (ACA) aimed to expand access to health care through improved health insurance coverage options to individuals and families with incomes near the national poverty levels.^1^ Following the implementation of the ACA in 2014, 40 states and the District of Columbia adopted the Medicaid expansion provision of the ACA so far, and 10 states chose not to expand Medicaid, creating a natural experiment for evaluating whether and to what extent Medicaid expansion affects health care access and health outcomes. Previous studies have shown that individuals residing in expansion states who receive a new diagnosis of cancer are more likely to have health insurance coverage, present with early disease stage at the time of diagnosis, and have better overall survival than individuals residing in nonexpansion states.^2,3,4,5,6,7^

Lung cancer is the second most commonly diagnosed cancer in the US and the leading cause of cancer-related mortality.^8^ Medicaid expansion may improve cancer survival through earlier detection (which is subject to lead-time bias), earlier stage at diagnosis, or improved access to care. While 1 study estimated the contribution of early stage at diagnosis to survival improvements in patients with non–small cell lung cancer (NSCLC),^9^ most previous studies that reported an association between Medicaid expansion and improved survival rate were subject to lead-time bias,^10,11^ and no studies have evaluated the association between Medicaid expansion and NSCLC mortality in a postoperative patient population for whom access to care is crucial for survival.

For individuals diagnosed with NSCLC confined to the lung and regional lymph nodes, surgical resection has historically been associated with the best prognosis and thus is currently recommended by evidence-based guidelines.^12^ The surgical removal of a pulmonary lobe (lobectomy) or entire lung (pneumonectomy) is technically complex to perform^13,14^ and challenging for patients to recover from. Postoperative complication rates are higher than many other oncologic surgical procedures, with 40% of patients experiencing an adverse event within 30 days of surgery for treatment of lung cancer.^13,14,15,16^ While in-hospital mortality is mainly associated with patients’ age and comorbidities,^17^ access to care is a major factor associated with deaths occurring after hospital discharge.^18,19,20^ Thus, access to health insurance coverage through Medicaid expansion may be especially relevant in this population for whom access to care during recovery following hospital discharge after lung cancer surgery is vital.

There are several mechanisms through which Medicaid expansion may be associated with improved survival among patients undergoing NSCLC surgical resection. First, Medicaid enrollees may be more likely to receive better management of health care conditions,^21,22,23,24^ including lung cancer comorbidities, such as cardiovascular and pulmonary diseases, for which access to care management is improved with Medicaid expansion.^25^ Poor management of those conditions is associated with worse survival.^26^ Second, insured individuals are less likely to delay seeking care,^27,28^ which is especially important during the first months of recovery from major cancer surgery, when patients are vulnerable to postoperative complications and have increased health care needs. This study evaluated the association between Medicaid expansion and postoperative mortality among patients undergoing surgical resection of stage I to III NSCLC.

## Methods

This cohort study used data from the National Cancer Database (NCDB), a nationwide hospital-based cancer registry jointly sponsored by the American Cancer Society and the American College of Surgeons, which includes over 70% of all patients newly diagnosed with cancer in the US.^29^ This study was granted an exemption from review and the requirement to obtain informed consent was waived by the Institutional Review Board of the Morehouse School of Medicine in Atlanta, Georgia, because deidentified data were used and no new data were collected. This study followed the Strengthening the Reporting of Observational Studies in Epidemiology (STROBE) reporting guideline for cohort studies.

Patients 45 to 64 years of age at the time of diagnosis—ages when individuals are not age-eligible for Medicare (individuals become age-eligible for Medicare coverage in the US at 65 years) and are most likely to be diagnosed with and die of NSCLC^30^—who were treated with curative lobectomy or pneumonectomy for stage I to III NSCLC in 27 states that expanded Medicaid eligibility by 2014 (Arizona, Arkansas, California, Colorado, Connecticut, Delaware, District of Columbia, Hawaii, Illinois, Iowa, Kentucky, Maryland, Massachusetts, Michigan, Minnesota, Nevada, New Hampshire, New Jersey, New Mexico, New York, North Dakota, Ohio, Oregon, Rhode Island, Vermont, Washington, and West Virginia) and 16 states that did not expand Medicaid eligibility by 2019 (Alabama, Florida, Georgia, Kansas, Mississippi, Missouri, Nebraska, North Carolina, Oklahoma, South Carolina, South Dakota, Tennessee, Texas, Utah, Wisconsin, and Wyoming) were selected from the NCDB.

Similar to a previous study evaluating the associations between Medicaid expansion and surgical outcomes, we excluded patients with other types of health insurance coverage (ie, only patients with Medicaid or no health insurance coverage were included).^31^ In-hospital mortality was defined as death date equal to the date of discharge; 30-day mortality was defined as death within 30 days from date of surgery among patients discharged from the hospital alive; and 90-day mortality was defined as death within 90 days from date of surgery among patients discharged from the hospital alive. Patients who were missing a surgery date, resided in states that expanded Medicaid between 2015 and 2019 (Alaska, Idaho, Indiana, Louisiana, Maine, Montana, Pennsylvania, and Virginia) or underwent surgery during the first 6 months after ACA expansion were excluded (as part of a washout period) (Figure).

**Figure.**
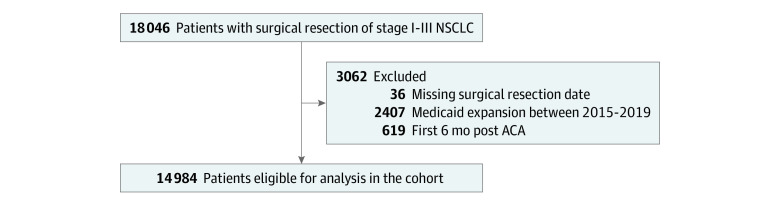
Study Sample Selection Selection among individuals 45 to 64 years of age who were newly diagnosed with stage I to III non–small cell lung cancer (NSCLC) in the National Cancer Database between 2004 and 2019. ACA indicates Patient Protection and Affordable Care Act.

### Statistical Analysis

Descriptive statistics were used to compare study population characteristics by Medicaid expansion status of patients’ state of residence. We tested the parallel trend assumption of difference-in-differences using linear generalized estimating equation models.^32^ After confirming parallel trends in outcomes between Medicaid expansion and nonexpansion states, we used a quasi-experimental design and difference-in-differences analyses to evaluate differences in postoperative mortality before the ACA (surgery between 2008 and 2013) and after the ACA (surgery between the third quarter of 2014 and 2019) by Medicaid expansion status. Models were adjusted for patients’ characteristics, including age and sex.

In sensitivity analyses, we used difference-in-differences analyses to evaluate differences in postoperative mortality before and after the ACA by Medicaid expansion status among individuals aged 18 to 64 years who underwent NSCLC surgery. We also used difference-in-differences analyses to evaluate differences in stage at diagnosis (stage I vs stage II-III) and comorbidities (identified according to the modified Charlson-Deyo Comorbidity Index)^33^ among individuals aged 45 to 64 years who underwent NSCLC surgery in expansion and nonexpansion states before and after the ACA.

We were able to access data on patients’ state of residence and NCDB reporting facility and adjusted all models for state and facility as random effects. Descriptive statistics included race and ethnicity categories as provided in the NCDB to demonstrate the diversity of the study population. We chose not to include race and ethnicity in the adjusted models to avoid incorrectly assigning race (a social construct that should only be used as a proxy for exposure to racism) as a risk factor.^34^ While certified tumor registrars follow highly standardized methods for extracting race and ethnicity information from medical records into the NCDB, strategies to record race and ethnicity information into medical records at each reporting facility may vary.

All analyses were conducted in SAS 9.4 (SAS Institute Inc). The statistical significance level for χ^2^ and Wald χ^2^ tests was set at .05; all tests were 2-sided. The analysis was conducted between March 28, 2021, and September 1, 2023.

## Results

The mean (SD) age of 14 984 individuals who underwent surgical resection of NSCLC was 56.3 (5.1) years, 54.6% were women, 45.4% were men, and most individuals were non-Hispanic Black (19.1%) or non-Hispanic White (69.2%) and lived in Medicaid expansion states (62.1%). Patients residing in nonexpansion states at the time of diagnosis more often were younger, racialized as non-Hispanic Black, uninsured, diagnosed with stage II disease, and had comorbidities compared with individuals residing in expansion states (Table 1). Before the ACA, there were no differences in the slopes between expansion and nonexpansion states for in-hospital mortality (β = 0; *P* = .83 and β = 0; *P* = .94; difference between slopes, *P* = .09), postdischarge 30-day mortality (β = 0.05; *P* = .05 and β = 0.03; *P* = .21; difference between slopes, *P* = .22), or postdischarge 90-day mortality (β = 0.04; *P* = .21 and β = 0; *P* = .90; difference between slopes, *P* = .13).

**Table 1.** Characteristics of Patients Included in the Study by Medicaid Expansion Status in State of Residence Before and After the ACA, National Cancer Database, 2008 to 2019^a^

Characteristic	Patients, No. (%)^b^					
	Total		Before the ACA		After the ACA	
	Nonexpansion (n = 5679)	Expansion (n = 9305)	Nonexpansion (n = 3072)	Expansion (n = 4218)	Nonexpansion (n = 2607)	Expansion (n = 5087)
Age group at diagnosis, y						
45-54	2136 (37.6)	3090 (33.2)	1296 (42.2)	1673 (39.7)	840 (32.2)	1417 (27.9)
55-64	3543 (62.4)	6215 (66.8)	1776 (57.8)	2545 (60.3)	1767 (67.8)	3670 (72.1)
Sex						
Male	2604 (45.9)	4201 (45.1)	1436 (46.7)	1968 (46.7)	1168 (44.8)	2233 (43.9)
Female	3075 (54.1)	5104 (54.9)	1636 (53.3)	2250 (53.3)	1439 (55.2)	2854 (56.1)
Race and ethnicity						
Hispanic	299 (5.3)	553 (6.0)	150 (4.9)	240 (5.7)	149 (5.7)	313 (6.2)
Indigenous populations^c^	27 (0.5)	40 (0.4)	14 (0.5)	16 (0.4)	13 (0.5)	24 (0.5)
Non-Hispanic Asian and Pacific Islander	69 (1.2)	587 (6.4)	36 (1.2)	215 (5.1)	33 (1.3)	372 (7.4)
Non-Hispanic Black	1293 (22.9)	1566 (16.9)	704 (23.0)	774 (18.5)	589 (22.7)	792 (15.7)
Non-Hispanic White	3940 (69.7)	6431 (69.6)	2138 (70.0)	2920 (69.8)	1802 (69.4)	3511 (69.4)
Other and missing^d^	51 (0.9)	128 (1.4)	30 (1.0)	53 (1.3)	21 (0.8)	75 (1.5)
Insurance						
Uninsured	2316 (40.8)	1277 (13.7)	1335 (43.5)	1023 (24.3)	981 (37.6)	254 (5.0)
Medicaid	3363 (59.2)	8028 (86.3)	1737 (56.5)	3195 (75.7)	1626 (62.4)	4833 (95.0)
Cancer stage						
I	3253 (57.3)	5452 (58.6)	1788 (58.2)	2407 (57.1)	1465 (56.2)	3045 (59.9)
II	1463 (25.8)	2232 (24.0)	765 (24.9)	1028 (24.4)	698 (26.8)	1204 (23.7)
III	963 (17.0)	1621 (17.4)	519 (16.9)	783 (18.6)	444 (17.0)	838 (16.5)
Comorbidities						
0	2892 (50.9)	4935 (53.0)	1522 (49.5)	2115 (50.1)	1370 (52.6)	2820 (55.4)
1	1949 (34.3)	2998 (32.2)	1142 (37.2)	1506 (35.7)	807 (31.0)	1492 (29.3)
≥2	838 (14.8)	1372 (14.7)	408 (13.3)	597 (14.2)	430 (16.5)	775 (15.2)
Facility						
NCI designated	590 (10.9)	1323 (14.8)	323 (11.3)	570 (14.5)	267 (10.4)	753 (15.0)
Comprehensive	2031 (37.5)	2272 (25.3)	1100 (38.6)	1008 (25.6)	931 (36.2)	1264 (25.1)
Teaching	1313 (24.2)	2674 (29.8)	655 (23.0)	1202 (30.5)	658 (25.6)	1472 (29.3)
Community	329 (6.1)	616 (6.9)	187 (6.6)	306 (7.8)	142 (5.5)	310 (6.2)
Other	1155 (21.3)	2082 (23.2)	583 (20.5)	853 (21.7)	572 (22.3)	1229 (24.4)

Abbreviations: ACA, Patient Protection and Affordable Care Act; NCI, National Cancer Institute.

^a^ Patients with surgery for non–small cell lung cancer between 2008 and 2019 were included, and patients who received surgery in the first 6 months after implementation of Medicaid expansion through the ACA were excluded.

^b^ Groups may not sum to totals due to missing data.

^c^ Indigenous populations includes all Indigenous populations of the Western hemisphere.

^d^ Other race and ethnicity category provided in the National Cancer Database was combined with missing race and ethnicity data.

In Medicaid expansion states, 30-day postoperative mortality decreased from 0.97% before the ACA to 0.26% after the ACA (0.71 percentage point decrease; *P* < .001). In contrast, the corresponding percentages remained unchanged in nonexpansion states (0.75% before the ACA and 0.68% after the ACA; *P* = .74), leading to a significant difference-in-differences of −0.64 percentage points (95% CI, −1.19 to −0.08 percentage points; *P* = .03).

Similarly, 90-day postoperative mortality decreased from 2.63% before the ACA to 1.32% after the ACA (1.31 percentage point decrease; *P* < .001) in Medicaid expansion states, with no significant change in nonexpansion states (2.43% before the ACA and 2.20% after the ACA; *P* = .57, leading to a significant difference-in-differences of −1.08 percentage points (95% CI, −2.08 to −0.08 percentage points; *P* = .03).

Although in-hospital postoperative mortality also decreased significantly in Medicaid expansion states, from 1.41% before the ACA to 0.77% after the ACA (0.63 percentage point decrease; *P* = .004); with no significant change in nonexpansion states (1.49% before the ACA and 1.20% after the ACA; 0.30 percentage point decrease), there was no significant difference-in-differences (*P* = .34) between expansion and nonexpansion states (Table 2).

**Table 2.** Pre- and Post-ACA DID for Postoperative Mortality After Non–Small Cell Lung Cancer Resection Among Patients 45 to 64 Years of Age Residing in Medicaid Expansion vs Nonexpansion States, National Cancer Database, 2008 to 2019^a^

Mortality	Nonexpansion states				Expansion states				Unadjusted		Adjusted^b^	
	ACA, %		Difference (95% CI)	*P* value	ACA, %		Difference (95% CI)	*P* value	DID (95% CI)	*P* value	DID (95% CI)	*P* value
	Before	After			Before	After						
In-hospital	1.49	1.20	0.30 (−0.26 to 0.86)	.29	1.41	0.77	0.63 (0.20 to 1.07)	.004	−0.34 (−1.05 to 0.37)	.34	−0.34 (−1.05 to 0.37)	.34
30 d	0.75	0.68	0.07 (−0.39 to 0.51)	.74	0.97	0.26	0.71 (0.37 to 1.04)	<.001	−0.64 (−1.19 to −0.08)	.03	−0.63 (−1.19 to −0.07)	.03
90 d	2.43	2.20	0.23 (−0.57 to 1.02)	.57	2.63	1.32	1.31 (0.70 to 1.92)	<.001	−1.08 (−2.08 to −0.08)	.03	−1.07 (−2.07 to −0.07)	.03

Abbreviations: ACA, Patient Protection and Affordable Care Act; DID, difference-in-differences.

^a^ Patients with surgery for non–small cell lung cancer between 2008 and 2013 (before the ACA) and between 2014 and 2019 (after the ACA) were included, and patients treated in the first 6 months after ACA were excluded.

^b^ Adjusted model included age and sex.

Results were similar in a sensitivity analysis that included individuals aged 18 to 64 years who underwent NSCLC surgery in expansion and nonexpansion states (eTable 1 in Supplement 1). Sensitivity analyses also showed no significant difference-in-differences results in stage at diagnosis or prevalence of comorbidities among individuals who received NSCLC surgery between expansion and nonexpansion states (eTable 2 in Supplement 1).

## Discussion

In this cohort study, we found that Medicaid expansion was associated with significant decreases in 30-day and 90-day postoperative mortality among patients who were discharged from the hospital following surgical resection of NSCLC, with no difference among individuals residing in nonexpansion states. In-hospital mortality following surgery did not differ in Medicaid expansion and nonexpansion states. Our findings suggest the importance of Medicaid expansion in improving access to care following hospital discharge.

Prior research has shown that Medicaid expansion is associated with increased health insurance coverage,^3^ and better access to care,^27,35,36,37,38^ especially among low-income populations.^28,37,39^ Previous studies have reported greater improvement in cancer survival among individuals diagnosed as having NSCLC residing in Medicaid expansion states than in nonexpansion states after ACA implementation,^6,10,11^ but these studies were subject to lead-time bias and were unable to distinguish between changes in patients’ previous health status (healthier individuals enrolling in Medicaid postexpansion) and improved access to care.

Similar to previous studies,^10,40,41,42^ our study focused on individuals 45 to 64 years of age, who are at higher risk of being diagnosed as having and dying of NSCLC cancer,^30^ are not age-eligible for Medicare, and often lack health insurance coverage as a result of restricted employment opportunities and limited affordable health insurance options.^40,41^ Unlike previous studies evaluating the association between Medicaid expansion and cancer survival from date of diagnosis,^10,11^ our study focused on early mortality after surgery. Therefore, our study is not subject to lead-time bias arising from improved access to diagnostic services. Furthermore, evaluating in-hospital mortality, postdischarge 30-day mortality, and postdischarge 90-day mortality separately enabled us to better control for patients’ previous health status (ie, patients had to be healthy enough to undergo surgery and to be discharged from the hospital) and addressed potential selection bias due to changes in group composition through time (ie, if healthier individuals enrolled in Medicaid after expansion). The lack of significant difference-in-differences results in stage of diagnosis and comorbidities between individuals who received surgery in expansion states and in nonexpansion states before and after the ACA further corroborates this strength of the present study.

Although all patients included in our study had access to surgical treatment according to evidence-based guidelines,^12^ uninsured patients may experience barriers in access to postoperative care after discharge from the hospital, including follow-up visits, pain management, rehabilitation, and access to prescription medications. In some studies, Medicaid expansion was associated with reduction in cost-related delays in needed specialist or follow-up care,^36,43^ and among trauma patients, ACA implementation was associated with gains in access to postdischarge care.^44^ Future studies should evaluate receipt of postdischarge care and estimate number of deaths averted in expansion states relative to nonexpansion states.

### Strengths and Limitations

The strengths of this study include nationwide data with information about individuals who received new diagnoses of NSCLC and their care 6 years after ACA implementation, with early mortality defined as in-hospital, 30 days after surgery date, and 90 days after surgery date, eliminating lead-time bias and composition bias, as patients had to be healthy enough to undergo surgery and be discharged from the hospital after surgery (for 30-day and 90-day mortality rate estimates). Lack of significant difference-in-differences results for stage at diagnosis and comorbidities between expansion and nonexpansion states before and after the ACA corroborated this strength (ie, no composition bias due to better health status prior to surgery in expansion states after the ACA). Additionally, we were able to control for state- and facility-level random effects, which is important given the heterogeneity in population characteristics between different states and treating facilities and the heterogeneity in Medicaid benefits offered by different states.

This study has several limitations, including unavailability of information about type of health insurance coverage prior to cancer diagnosis, receipt of postoperative care, and cause of death, as these data are not available in the NCDB. Additionally, comorbidities recorded in the medical record may not fully reflect patient health status. Although the NCDB captures over 70% of individuals newly diagnosed with cancer in the US, it is not population-based. Therefore, certain populations that are less likely to be treated at Commission on Cancer–accredited facilities (patients residing in low-income or rural areas) may be underrepresented in the NCDB for some states.^45^ Nonetheless, cancer cases captured by the NCDB closely resemble those of population-based cancer registries.^29^ Finally, it is possible that the quality of care provided by the NCDB facilities located in expansion and nonexpansion states differs. However, this difference was examined previously, and there do not appear to be large differences in these 2 cohorts of hospitals.^46^ Nonetheless, we controlled for both state and facility as random effects in our models.

## Conclusions

In this cohort study, Medicaid expansion after implementation of the ACA was associated with improved 30-day and 90-day postoperative survival among patients discharged from the hospital following surgical resection of NSCLC. As policymakers consider whether to expand Medicaid or change different ACA provisions, these findings provide important evidence of the positive health consequences associated with coverage expansion.

## Notes

Supplement 1. **eTable 1.** Sensitivity Analysis of Pre- and Post-ACA Difference-in-Differences Postoperative Mortality Among Individuals Aged 18-64 Years After NSCLC Resection in Medicaid Expansion and Non-Expansion States, NCDB 2008-2019 **eTable 2.** Sensitivity Analyses of Pre- and Post-ACA Difference-in-Differences for Stage at Diagnosis and Comorbidities in Medicaid Expansion and Non-Expansion States, NCDB 2008-2019

Supplement 2.
Data Sharing Statement
