# Supplementary material for: Medicaid Expansion Under the Affordable Care Act and Early Mortality Following Lung Cancer Surgery
Source: JAMA Netw Open. 2024 Jan 12;7(1):e2351529. doi: 10.1001/jamanetworkopen.2023.51529 (PMC10787311; doi:10.1001/jamanetworkopen.2023.51529)
Supplement: Supplement 1. — eTable 1. Sensitivity Analysis of Pre- and Post-ACA Difference-in-Differences Postoperative Mortality Among Individuals Aged 18-64 Years After NSCLC Resection in Medicaid Expansion and Non-Expansion States, NCDB 2008-2019 eTable 2. Sensitivity Analyses of Pre- and Post-ACA Difference-in-Differences for Stage at Diagnosis and Comorbidities in Medicaid Expansion and Non-Expansion States, NCDB 2008-2019 [file jamanetwopen-e2351529-s001.pdf]

Supplementary Online Content

Nogueira LM, Boffa DJ, Jemal A, Han X, Yabroff KRK. Medicaid expansion under the Affordable Care Act and early mortality following lung cancer surgery. *JAMA Netw Open*. 2023;7(1):e2351529. doi:10.1001/jamanetworkopen.2023.51529

eTable 1. Sensitivity Analysis of Pre- and Post-ACA Difference-in-Differences Postoperative Mortality Among Individuals Aged 18-64 Years After NSCLC Resection in Medicaid Expansion and Non-Expansion States, NCDB 2008-2019

eTable 2. Sensitivity Analyses of Pre- and Post-ACA Difference-in-Differences for Stage at Diagnosis and Comorbidities in Medicaid Expansion and Non-Expansion States, NCDB 2008-2019

This supplementary material has been provided by the authors to give readers additional information about their work.

© 2024 Nogueira LM et al. *JAMA Network Open*.

eTable 1. Sensitivity analysis of pre- and post-ACA difference-in-differences postoperative mortality among individuals aged 18-64 years after NSCLC resection in Medicaid expansion and non-expansion states, NCDB 2008-2019

	Non-Expansion States				Expansion States							
	ACA				ACA				Unadjusted		Adjusted	
Mortality	Pre	Post	Difference	P-value	Pre	Post	Difference	P-value	DID (95% CI)	P-value	DID (95% CI)	P-value
In-hospital	1.43	1.12	0.30 (-0.21, 0.83)	0.2	1.34	0.72	0.61 (0.20, 1.02)	0.003	-0.30 (-0.97, 0.36)	0.3	-0.31 (-0.97, 0.35)	0.3
30 day	0.74	0.65	0.08 (-0.33, 0.50)	0.7	0.88	0.26	0.62 (0.30, 0.94)	<.0001	-0.54 (-1.06, -0.02)	0.04	-0.54 (-1.06, -0.02)	0.04
90 day	2.36	2.11	0.25 (-0.50, 0.99)	0.5	2.44	1.26	1.16 (0.60, 1.74)	<.0001	-0.92 (-1.85, -0.02)	0.04	-0.92 (-1.86, -0.02)	0.04

Notes: ACA = Affordable Care Act, DID = Difference in Differences
Medicaid Expansion States included AZ, AR, CA, CO, CT, DE, DC, HI, IL, IA, KY, MD, MA, MI, MN, NV, NH, NJ, NM, NY, ND, OH, OR, RI, VT, WA, WV and non-expansion states included AL, FL, GA, KS, MS, MO, NE, NC, OK, SC, SD, TN, TX, UT, VA, WI. Patients who received surgery for non-small cell lung cancer (NSCLC) between 2008-2013 (Pre-ACA) and 2014-2019 (Post-ACA) were included. Patients treated in the first 6 months post-ACA were excluded.
Adjusted model included age and sex.

© 2024 Nogueira LM et al. *JAMA Network Open*.

eTable 2. Sensitivity analyses of pre- and post-ACA difference-in-differences for stage at diagnosis and comorbidities in Medicaid expansion and non-expansion states, NCDB 2008-2019

	Non-Expansion				Expansion							
	ACA				ACA				Unadjusted		Adjusted	
Mortality	Pre	Post	Difference	P-value	Pre	Post	Difference	P-value	DID (95% CI)	P-value	DID (95% CI)	P-value
Stage I at diagnosis	58.5	57.2	1.36 (-1.39, 4.10)	0.3	58.4	60.3	-1.93 (-4.04, 0.17)	0.07	3.29 (-0.17, 6.75)	0.06	3.07 (-0.37, 6.51)	0.08
≥1 Comorbidity	52.2	49.6	2.57 (-0.16, 5.3)	0.06	51.3	45.7	5.67 (3.56, 7.78)	<.0001	-3.09 (-6.54, -0.36)	0.08	-3.25 (-6.69, -0.20)	0.06

Notes: ACA = Affordable Care Act, DID = Difference in Differences
Medicaid Expansion States included AZ, AR, CA, CO, CT, DE, DC, HI, IL, IA, KY, MD, MA, MI, MN, NV, NH, NJ, NM, NY, ND, OH, OR, RI, VT, WA, WV and non-expansion states included AL, FL, GA, KS, MS, MO, NE, NC, OK, SC, SD, TN, TX, UT, VA, WI. Patients who received surgery for non-small cell lung cancer (NSCLC) between 2008-2013 (Pre-ACA) and 2014-2019 (Post-ACA) were included. Patients treated in the first 6 months post-ACA were excluded.
Adjusted model included age and sex.

© 2024 Nogueira LM et al. *JAMA Network Open*.
