# Supplementary material for: Medicaid Expansion Under the Affordable Care Act and Early Mortality Following Lung Cancer Surgery
Source: JAMA Netw Open. 2024 Jan 12;7(1):e2351529. doi: 10.1001/jamanetworkopen.2023.51529 (PMC10787311; doi:10.1001/jamanetworkopen.2023.51529)
Supplement: Supplement 2. — Data Sharing Statement [file jamanetwopen-e2351529-s002.pdf]

# Data Sharing Statement

Nogueira. Medicaid Expansion Under the Affordable Care Act and Early Mortality Following Lung Cancer Surgery. *JAMA Netw Open*. Published January 12, 2024. doi:10.1001/jamanetworkopen.2023.51529

**Data**
**Data available:** No

**Additional Information**
**Explanation for why data not available:** As per Commission on Cancer data use agreement, National Cancer Database data is only available to researchers affiliated with accredited institutions.
